# CPT-11 converting carboxylesterase and topoisomerase I activities in tumour and normal colon and liver tissues

**DOI:** 10.1038/sj.bjc.6690364

**Published:** 1999-05-01

**Authors:** S Guichard, C Terret, I Hennebelle, I Lochon, P Chevreau, E Frétigny, J Selves, E Chatelut, R Bugat, R Canal

**Affiliations:** Groupe de Pharmacologie Clinique et Expérimentale, Institut Claudius Regaud, 20–24 rue de Pont Saint Pierre 31052, Toulouse Cedex, France; Clinique du Parc, 4 rue Mespoul, 31300 Toulouse, France; Clinique Sarrus, 49, allées Charles de Fitte, 31300 Toulouse, France; Service d'Anatomie Pathologique, Centre Hospitalier Universitaire Purpan, Toulouse, France; Université Paul Sabatier, 118 Route de Narbonne, Toulouse, France

## Abstract

CPT-11 is a prodrug activated by carboxylesterases to the active metabolite SN-38 which is a potent inhibitor of topoisomerase I. CPT-11 is of clinical interest in the treatment of colorectal cancer. We evaluated the activities of CPT-11 converting carboxylesterase (CPT-CE) and topoisomerase I (topo I) in 53 colorectal tumours, in eight liver metastases and in normal tissue adjacent to the tumours. Both CPT-CE and topo I activities were widely variable in the malignant and the normal tissue of patients with colorectal carcinomas. CPT-CE was only two to threefold lower in primary tumours compared to normal liver, suggesting that a local conversion to SN-38 might occur in tumour cells. CPT-CE was similar in liver and in normal colon tissues. Levels of topo I in tumour ranged from 580 to 84 900 U mg protein^−1^ and was above 40 000 U mg protein^−1^ in 11 of 53 patients. Similarly, a very high ratio (> 5) between tumour and normal tissues were observed in 12 of 53 patients. An inverse correlation was observed between the topo I activity and the clinical stage of disease. Clinical studies are in progress in our institution to explore a possible relationship between CPT-CE and topo I activities in tumour cells and the response to CPT-11-based chemotherapy in patients with colorectal cancer. © 1999 Cancer Research Campaign


*British Journal of Cancer* (1999) **80**(3/4), 364–370
© 1999 Cancer Research Campaign
Article no. bjoc.1998.0364

### CPT-11 converting carboxylesterase and topoisomerase I activities in tumour and normal colon and liver tissues

**S Guichard[1], C Terret[1], I Hennebelle[1], I Lochon[1], P Chevreau[2], E Frétigny[3], J Selves[4], E Chatelut[1,5], R Bugat[1,5] and P Canal[1]**

[1]Groupe de Pharmacologie Clinique et Expérimentale, Institut Claudius Regaud, 20–24 rue de Pont Saint Pierre 31052, Toulouse Cedex, France; [2]Clinique du Parc, 4 rue Mespoul, 31300 Toulouse, France; [3]Clinique Sarrus, 49, allées Charles de Fitte, 31300 Toulouse, France; [4]Service d'Anatomie Pathologique, Centre Hospitalier Universitaire Purpan, Toulouse, France; [5]Université Paul Sabatier, 118 Route de Narbonne, Toulouse, France

**Summary** CPT-11 is a prodrug activated by carboxylesterases to the active metabolite SN-38 which is a potent inhibitor of topoisomerase I. CPT-11 is of clinical interest in the treatment of colorectal cancer. We evaluated the activities of CPT-11 converting carboxylesterase (CPT-CE) and topoisomerase I (topo I) in 53 colorectal tumours, in eight liver metastases and in normal tissue adjacent to the tumours. Both CPT-CE and topo I activities were widely variable in the malignant and the normal tissue of patients with colorectal carcinomas. CPT-CE was only two to threefold lower in primary tumours compared to normal liver, suggesting that a local conversion to SN-38 might occur in tumour cells. CPT-CE was similar in liver and in normal colon tissues. Levels of topo I in tumour ranged from 580 to 84 900 U mg protein$^{-1}$ and was above 40 000 U mg protein$^{-1}$ in 11 of 53 patients. Similarly, a very high ratio (> 5) between tumour and normal tissues were observed in 12 of 53 patients. An inverse correlation was observed between the topo I activity and the clinical stage of disease. Clinical studies are in progress in our institution to explore a possible relationship between CPT-CE and topo I activities in tumour cells and the response to CPT-11-based chemotherapy in patients with colorectal cancer.

Irinotecan, 7-ethyl-10[4-(1-piperidino)-1-piperidino]carbonyloxy-camptothecin (CPT-11), is a topoisomerase I (topo I) inhibitor commonly used in the treatment of colorectal tumours, and promising results have been recently reported in metastatic disease (Rothenberg et al, 1996; Rougier et al, 1997). CPT-11 is a prodrug, which differs structurally from other camptothecin derivatives by a bulky piperidino side chain located at the C-10 position of the camptothecin molecule (Kunimoto et al, 1987). This piperidino group must be cleaved enzymatically by a carboxylesterase to form SN-38, which is the active metabolite (Tanizawa et al, 1994). The anti-tumour activity of CPT-11 is, therefore, dependent on both its activation by the CPT-11-converting carboxylesterase (CPT-CE) and the cellular level of the target enzyme topo I.

Carboxylesterases are a family of ubiquitary enzymes that react with many substrates such as p-nitrophenyl acetate (p-NPA) and nitrophenyl butyrate. They are present in vertebrates and classified by substrates for which they have high affinity and the specific compounds that inhibit their activity (Miller et al, 1980; Satoh & Hosokawa, 1995). The CPT-CE has been characterized in liver microsomes (Rivory et al, 1996; Slatter et al, 1997), and showed the relative inefficiency for CPT-11 transformation in the liver. In fact, the low efficiency of carboxylesterases seems to be a general feature of the human enzymes, and the interspecies comparison of the carboxylesterases with a panel of substrates, demonstrated that the human enzymes were among the less efficient (Hosokawa et al, 1990). However, a specific CPT-CE was isolated from rat serum (Tsuji et al, 1991). These authors demonstrated that the CPT-CE exhibited different enzymatic properties compared to the other carboxylesterases and that the Km was different between p-NPA and CPT-11. The conversion of CPT-11 into SN-38 has been studied in a wide variety of tissues, cell lines and purified enzyme preparations in vitro (Jansen et al, 1997; Kawato et al, 1991; Ogasawara et al, 1995; Rivory et al, 1996; Satoh et al, 1994; Slatter et al, 1997; Tsuji et al, 1991; van Ark-Otte et al, 1998). The sensitivity of proliferating tissues or cell lines to cytotoxic effects of CPT-11 may be related to their carboxylesterase levels (Kawato et al, 1991; Ogasawara et al, 1995). A decreased conversion of CPT-11 to SN-38 has been reported in vitro in resistant ovarian and non-small-cell lung cancer cell lines (Niimi et al, 1992; Ogasawara et al, 1995). However, little is known about CPT-CE activity in human tumours; the use of CPT-11 as a substrate in our study, insured the specific determination of CPT-CE among the overall carboxylesterases.

CPT-11 is a topo I inhibitor. Topo I is a nuclear enzyme that regulates the torsional strain of the DNA. Topo I enzymatic reaction involves the binding of topo I to DNA, the cleavage of one strand of DNA, the passage of the intact strand through topo I–DNA complex, and the resealing of the cleaved strand, without modification of the DNA sequence. SN-38 stabilizes the topo I–DNA complex or 'cleavable complex', thereby maintaining a single-strand DNA break. The collision between such an SN-38 stabilized cleavable complex and a DNA replication fork converts the single-strand DNA break to a double-strand break, which is highly deleterious (Creemers et al, 1994; Slichenmyer et al, 1993). The cytotoxicity of topo I inhibitors seems to be related to the level of topo I in cells: cells expressing high levels of topo I are hypersensitive to topo I inhibitors, while a decreased level of the target enzyme could be a factor of resistance to camptothecin derivatives

*Received 24 July 1998*
*Revised 21 September 1998*
*Accepted 6 November 1998*

*Correspondence to:* P Canal

(Knab et al, 1993; Benedetti et al, 1993; Takano et al, 1992). High topo I levels have been reported in colon (Giovanella et al, 1989; Husain et al, 1994; McLeod et al, 1994), prostate (McLeod et al, 1994), ovarian carcinomas (Codegoni et al, 1998; Cornarotti et al, 1996; van der Zee et al, 1994) and lymphomas (Potmesil et al, 1988). Several studies (Giovanella et al, 1989; Husain et al, 1994; McLeod et al, 1994) emphasized a positive ratio of topo I activity between tumour and adjacent normal tissue in colon carcinomas, associated with a wide interpatient variability.

In the current study, the activities of CPT-CE and topo I were measured simultaneously in the tumour and adjacent normal tissue of 59 patients with colorectal cancer to assess the interpatient variability, the possible correlations between the various tissues and the identification of demographic and pathological factors that influence drug activation and efficacy.

### MATERIALS AND METHODS

### Chemicals

CPT-11 and SN-38 were provided by Rhône Poulenc Rorer laboratories (Vitry sur Seine, France).

### Patients

Evaluation of CPT-CE and topo I activities was conducted in 59 patients undergoing surgery for colon or rectal carcinoma. The surgical procedure was for initial resection in the majority of patients (51 patients), while six patients underwent hepatic lobectomy for solitary colorectal metastases and two patients had simultaneous tumorectomy and partial hepatectomy. This study was conducted after obtaining informed consent from patients.

Immediately after resection, portions of non-necrotic tumour and adjacent normal tissue (> 5 cm from tumour) were excised by a pathologist and frozen in liquid nitrogen. Samples were stored at –80°C until analysis of enzyme activity. All analysis of frozen samples were performed within 2 months. We have verified that storage up to 6 months did not affect CPT-CE and topo I activities. Moreover, in seven tumours, enzymatic activities were determined in two independent portions of the resection to check the homogeneity in enzyme activities. No significant difference was observed.

The influence of tissue enzyme activities in response to CPT-11 therapy was not evaluated in this study because of the small number of individuals who received uniform post-operative therapy within each Dukes' stage.

### Analysis of enzyme activities

#### *CPT-CE activity*

Tumour and normal tissues were homogenized in 35 mM sodium phosphate buffer pH 7.5. The homogenate was centrifuged at 20 000 ***g*** for 30 min at 4°C. Cytosolic protein concentration was determined with the Bradford method (Bradford, 1976).

Preliminary study was made on pooled tumoural cytosols to optimize CPT-CE determination. Different concentrations in substrates (1–20 μM CPT-11), in protein concentrations (0.5–4 mg $ml^{-1}$) and incubation times (5–120 min) have been tested. A plateau of SN-38 formation was obtained after 30 min incubation; a linearity of the enzymatic reaction was observed as a function of the protein concentration in the presence of an excess in substrate (5 μM) (data not shown). Moreover, in case of very low (< 1 pmol $min^{-1}$ mg $protein^{-1}$) or very high activity (> 5 pmol $min^{-1}$ mg $protein^{-1}$) in tumours, a kinetic study (4 points) of the enzymatic activity was again performed with either a higher or a lower protein concentration.

The CPT-CE activity was then carried out by pre-incubating 80 μl of cytosolic proteins (3 mg $ml^{-1}$) for 5 min at 37°C in Eppendorf tubes and then 5 μM CPT-11 (20 μl) was added for an additional 60 min (Rivory et al, 1996). The reaction was stopped by addition of 100 μl of an ice-cold mixture of acetonitrile, water and 0.1 N hydrochloric acid (HCl) (3:3:3, by vol). After centrifugation at 4°C for 15 min at 400 ***g***, the supernatant (150 μl) was recovered and 50 μl aliquots were analysed for SN-38 concentration. SN-38 produced during the incubation was measured by the high-performance liquid chromatography (HPLC) method of Rivory and Robert (Rivory and Robert, 1994). Briefly, separation was performed on a Nucleosil C18 column (5 μm, 300 mm × 3.9 mm), eluted with a mobile phase consisting of 0.075 M ammonium acetate buffer (pH 6.4)-acetonitrile (60:40, v/v) containing tetrabutylammonium phosphate (PIC A Waters, Saint Quentin en Yvelines, France) at a final concentration of 5 mM. Detection of SN-38 was carried out with a Shimadzu fluorometer with excitation and emission wavelengths biased towards SN-38 detection at 380 and 540 nm respectively. Standards were prepared from a 100 μg $ml^{-1}$ stock solution of SN-38 diluted serially in a mixture of acetonitrile, water and 0.1 N HCl (3:3:3, by vol). Standard curves were constructed for each batch of samples and were linear from 2.5 to 25 pmol SN-38 $ml^{-1}$. CPT-CE activity was expressed as pmol $min^{-1}$ mg $protein^{-1}$.

#### *DNA topo I activity*

Tumour and normal tissues were homogenized in 0.01 M phosphate-buffered saline (PBS) buffer pH 7.4. Crude nuclear extracts were prepared as described previously by Deffie et al, (1989). Briefly, the homogenate was washed twice with cold nuclear buffer (NB) (2 mM $K_2HPO_4$, 5 mM magnesium chloride ($MgCl_2$), 150 mM sodium chloride (NaCl), 1 mM EDTA and 0.1 mM dithiothreitol (DTT), and resuspended in 1 ml of NB containing 0.35% of Triton X-100 and 1 mM phenylmethylsulphonyl fluoride (PMSF). It was kept on ice for 10 min, washed twice with cold NB. Nuclear proteins were extracted for 1 h at 4°C with cold NB containing 0.35 M NaCl. After centrifugation at 18 000 ***g*** for 10 min at 4°C, the supernatant was added with 50% glycerol. The protein concentration was determined using bicinchoninic acid (Smith et al, 1985).

The DNA topo I activity was determined according to Jaxel et al (1989) with the use of a standard curve of purified topo I (TopoGen Inc, Colombus, OH, USA). The reaction mixture contained 50 mM potassium chloride, 5 mM $MgCl_2$, 0.1 mM EDTA, 15 μg ml bovine serum albumin (BSA), 10 mM Tris-HCl pH 7.5, 0.5 mM DTT, 0.5 μg pKS plasmid, and either serial dilutions of nuclear extract or dilutions of purified topo I (0–5 U) in a final volume of 20 μl. After 10 min at 37°C, the reaction was stopped by addition of 1% sodium dodecyl sulphate (SDS), 20 mM EDTA, 0.5 mg $ml^{-1}$ proteinase K, and incubation was carried out for an additional 30 min. Dye solution (2.5 μl of 10 mM $Na_2HPO_4$, 0.3% bromophenol blue, 16% Ficoll) was then added to samples which were electrophoresed in 1% agarose gel in Tris borate EDTA migration buffer at 30 V overnight. Gel was stained with ethidium bromide and visualized in a UV transilluminator. Gel

© Cancer Research Campaign 1999

pictures were analysed by Photostyler and Optimas softwares. Topo I activity of samples was calculated from the standard curve of topo I established for each experiment. Results were expressed as units of topo I activity per mg protein.

### Statistical analysis

Statistical tests were performed after verification of the gaussian distribution of the population for the different parameters studied. Comparisons were carried out after controlling for homogeneity of the variances. The comparisons of averages used two-sided *t*-test, while the comparisons between the tumour and the normal tissue used paired *t*-tests. A $\chi^2$ test was performed to evaluate the distribution of patients within different subgroups. The correlation between two parameters was calculated and its significance was evaluated by a *t*-test. The significance level used for all tests was 0.05.

### RESULTS

### Characteristics of the population

Enzyme activities were assessed in tissue from 59 consecutive patients (22 male and 37 female). The median age of the patients was 71 years and ranged from 30 to 85. Liver metastases and adjacent normal liver were also obtained from eight patients. The tumours were preliminary Dukes' stage A ($n$ = 10), B ($n$ = 15) and C ($n$ = 28).

### CPT-CE activity

The distribution of CPT-CE activity in tumour and normal colon tissues is illustrated in Figure 1. CPT-CE activity was variable from one specimen to another with a coefficient of variation of about 50% in primary tumour and normal tissues. The mean values for CPT-CE activity were 2.24 pmol min$^{-1}$ mg$^{-1}$ protein in primary tumour tissue and 3.06 pmol min$^{-1}$ mg protein$^{-1}$ in normal colon tissue (Table 1). CPT-CE activity was significantly higher in normal tissue than in tumour tissue ($P$ = 0.0041) (Figure 2). In 43 out of 53 primary tumours, CPT-CE activity ranged between 1 and 3 pmol min$^{-1}$ mg protein$^{-1}$, whereas the variability was greater among normal tissue samples.

The ratio of CPT-CE activity between tumour and normal tissue was evaluated to assess the degree of tumour-specific CPT-11 activation (Figure 3). Eighteen of 53 patients had a ratio above 1 and among these 18 patients, 11 were suffering from Dukes' C disease. This distribution is significantly different from that observed in the two other Dukes' groups ($P$ = 0.016, $\chi^2$, ddl = 2).

The mean CPT-CE activity in liver metastases ($n$ = 8) was 1.90 pmol min$^{-1}$ mg protein$^{-1}$ (range 0.42–4.17), which is comparable to that observed in primary tumours (2.24) ($P$ = 0.414, dF = 59). CPT-CE activity in normal liver ($n$ = 8) ranged from 2.05 to 8.17 pmol min$^{-1}$ mg protein$^{-1}$ and this was significantly higher than in liver metastases ($P$ = 0.009) (Figure 2). Consequently, the ratio of CPT-CE activity between liver metastases and normal liver ranged from 0.19 to 1.02 (mean 0.47).

No difference was observed in CPT-CE activity between the normal liver and the normal colon ($P$ = 0.094; dF = 59) (Figure 2).

Tumour CPT-CE activities were independent of the age and the sex of the patient, the differentiation state of the tumour and the stage of the disease.

### Topo I activity

Mean, CV and median values of topo I activities in tumour and normal colon tissues are summarized in Table 1. The coefficients of variation in both normal and tumour tissues were high: 76% and 79% respectively. The distribution of topo I activity is illustrated in Figure 4. Levels of topo I in the tumour ranged from 580 to 84 900 U mg protein$^{-1}$. In 11 out of 53 tumours, topo I activity was greater than 40 000 U mg protein$^{-1}$. Topo I activity was significantly different in primary tumour compared to normal tissue ($P$ = 0.008, $n$ = 53). Moreover, the topo I activity was significantly lower in the liver metastases than in the normal liver ($P$ = 0.003, $n$ = 8). Finally, topo I activity was similar in normal liver and the normal colon ($P$ = 0.708, dF = 59).

The ratio between tumour and normal tissue activities was highly variable from one patient to another (Figure 5). Thirty-two

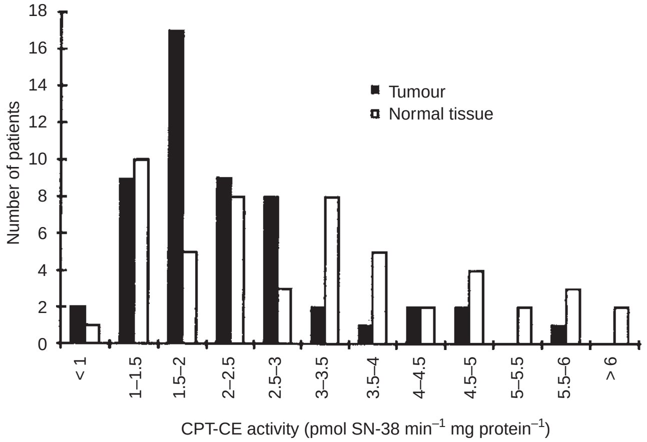

**Figure 1** Distribution of CPT-11 converting carboxylesterase (CPT-CE) activity in colon tumour and adjacent colon mucosa ($n$ = 53)

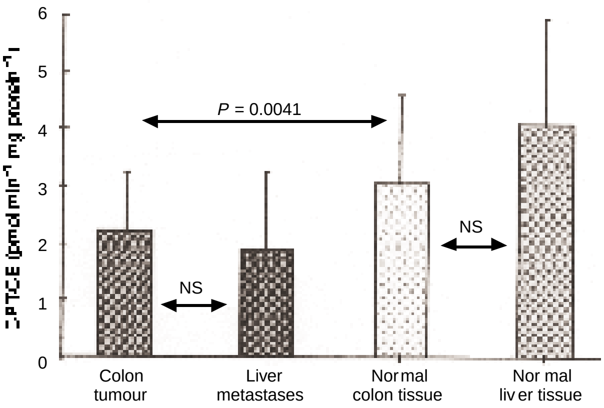

**Figure 2** Comparison of CPT-11 converting carboxylase (CPT-CE) activity in tumour and normal tissues. No difference between tumour colon tisssue and liver metastases or between colon and liver normal tissues. (NS) Significant difference between tumour and normal colon tissue (Student's test: $P$ = 0.0041)

© Cancer Research Campaign 1999

**Table 1** Enzyme activities in tumoural and normal colon and liver tissues

| | | Colon tumour *n* = 53 | Colon tissue *n* = 53 | Tumour vs normal tissue ratio | Liver metastases *n* = 8 | Normal liver *n* = 8 | Metastases vs normal tissue ratio |
|---|---|---|---|---|---|---|---|
| CPT-CE | Mean | 2.24 | 3.06 | 1.06 | 1.90 | 4.09 | 0.49 |
| pmol min$^{-1}$ | C.V. (%) | 46 | 51 | 98 | 73 | 45 | 68 |
| mg protein$^{-1}$ | Median | 1.81 | 2.93 | 0.74 | 1.48 | 3.47 | 0.42 |
| | Min | 0.76 | 0.74 | 0.17 | 0.42 | 2.05 | 0.19 |
| | Max | 5.77 | 7.33 | 5.77 | 4.17 | 8.17 | 1.02 |
| Topo I | Mean | 25291 | 17339 | 3.40 | 8500 | 19250 | 0.47 |
| U mg protein$^{-1}$ | C.V. (%) | 76 | 79 | 139 | 63 | 57 | 52 |
| | Median | 23200 | 17100 | 1.64 | 8700 | 15900 | 0.51 |
| | Min | 580 | 500 | 0.09 | 1400 | 8600 | 0.08 |
| | Max | 84900 | 64900 | 20 | 16800 | 36800 | 0.84 |

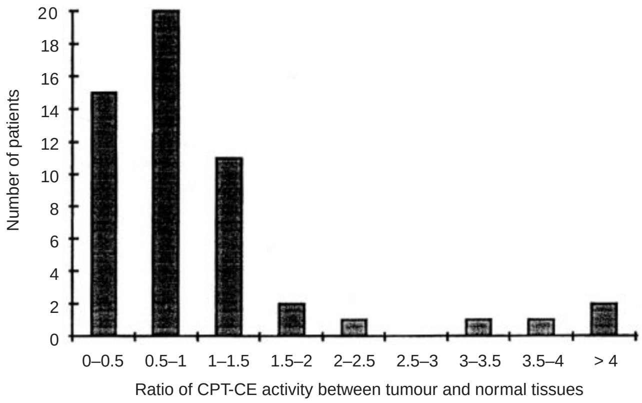

**Figure 3** Distribution of the ratio of CPT-11 converting carboxylesterase (CPT-CE) activity between tumour and adjacent colon mucosa (*n* = 53)

of 53 patients showed a ratio above 1, and in 12 cases, the ratio was above 5. So, a favourable gradient between the tumour and the normal colon would be achieved in more than half of the patients.

Tumour topo I activities were independent of the age and the sex of the patient, and the differentiation state of the tumour.

Figure 6 illustrates the distribution of topo I activity as a function of clinical stage of the disease (Dukes' or metastases). Topo I activity correlated inversely with clinical stage of the disease: the mean topo I activity was lower in stage C than in stage A tumours ($P = 0.05$, dF = 36). Moreover, in the Dukes' C tumours ($n = 28$), 12 samples had a low topo I activity (< 10 000 U mg protein$^{-1}$) while ten had a very high activity (> 35 000 U mg protein$^{-1}$), suggesting a bimodal distribution. Assuming that the presence of metastases is a step further in the progression of the disease, we compared topo I activity in primary tumour and in liver metastases. Topo I activity was significantly lower in liver metastases than in primary tumour ($P < 0.001$, dF = 59), and significantly lower in liver metastases than in Dukes' C subgroup ($P < 0.001$, dF = 34).

### DISCUSSION

This study provides the first simultaneous analysis of both the CPT-CE and the topo I activities in 53 patients with primary colorectal carcinomas and eight patients with liver metastases. Characterization of these enzymes in colorectal cancer is of clinical interest because of their roles in the regulation of activity and/or toxicity of CPT-11, one of the new agents active in colorectal cancer (Rothenberg et al, 1996; Rougier et al, 1997).

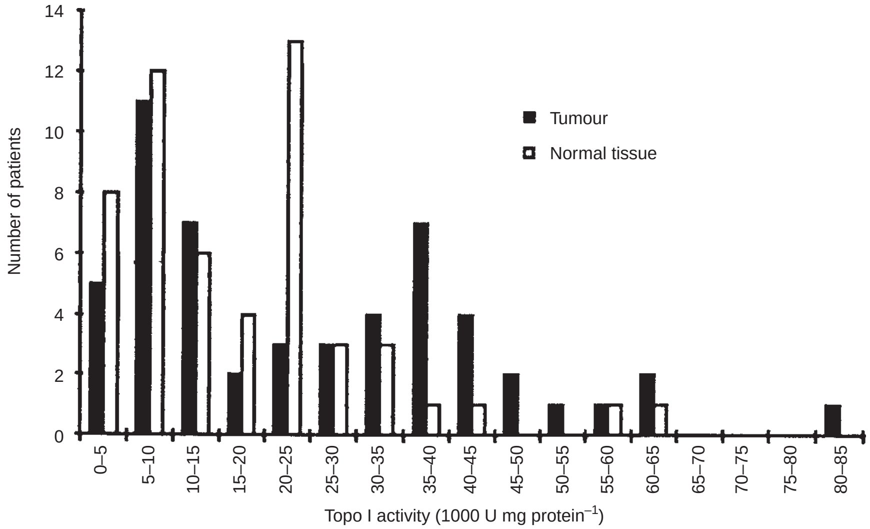

**Figure 4** Distribution of topoisomerase I (topo I) activity in colon tumour and adjacent colon mucosa (*n* = 53)

© Cancer Research Campaign 1999

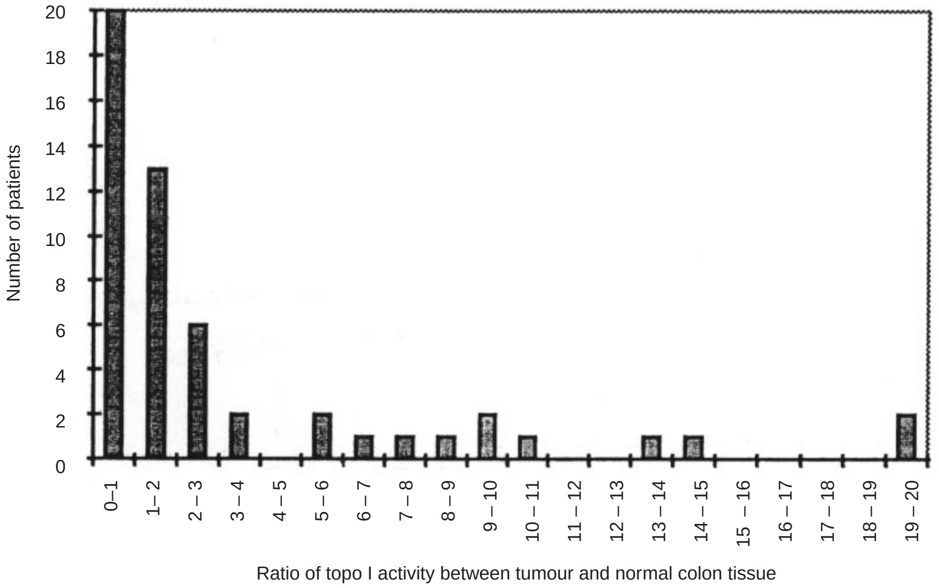

**Figure 5** Distribution of the ratio of topoisomerase I (topo I) activity between colon tumour and adjacent colon mucosa ($n$ = 53)

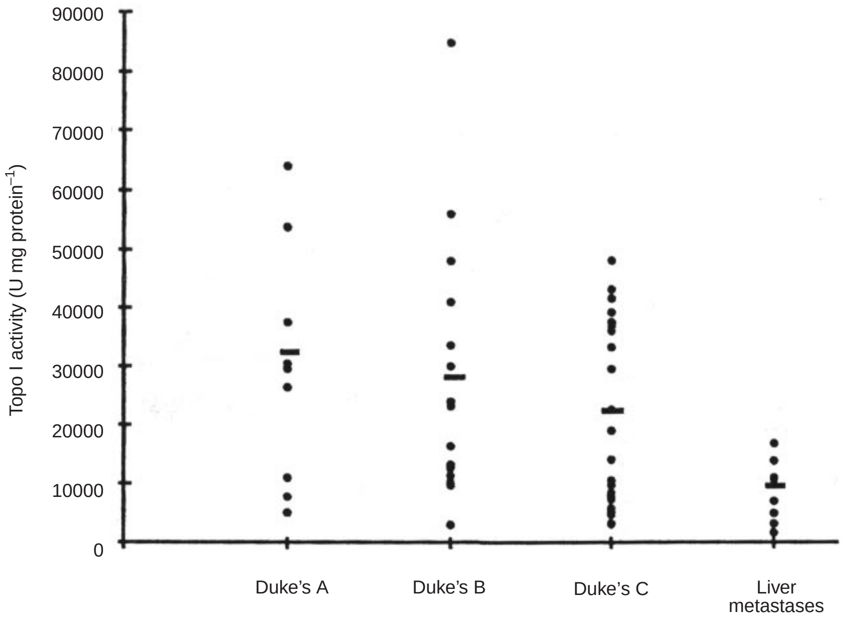

**Figure 6** Variation of topoisomerase I (topo I) activity as a function of tumour disease in colorectal tumours. Bars expressed means

To be active, CPT-11 must be converted by a carboxylesterase into SN-38, which is a potent inhibitor of the topo I (Tanizawa et al, 1994). Our study demonstrated an important variability in CPT-CE activity in the 53 primary tumour specimens and that CPT-CE activity was equal in primary and secondary colorectal tumours and was only about two- to three-fold lower than in human normal liver tissue. Although conversion of CPT-11 into SN-38 is likely to occur in the major pharmacological sites, i.e. the liver in humans, there may also be a local activation of CPT-11 in tumour tissue. The local activation of CPT-11 is of potential importance because of the different metabolic pathways between the hepatocytes and the tumour cells. In the liver, CPT-11 can be inactivated by cytochrome 3A4 in 7-ethyl-10[4-N-(5-aminopentanoic acid)-1-piperidino]carbonyloxy-camptothecin or APC (Haaz et al, 1998), which is not a substrate for human liver carboxylesterase (Rivory et al, 1996). Moreover, SN-38 produced in hepatocytes is glucuro-conjugated and thereby inactivated before excretion in the bile and urine (Gupta et al, 1994; Rivory & Robert, 1995). A very different metabolism occurs in a tumour cell. Neither the inactivation of the CPT-11 by cytochrome 3A4 nor the glucuro-conjugation is likely to occur in the tumour cells since the activity of these enzymes is very low in colon tumour cells (Massaad et al, 1993; McKay et al,

 © Cancer Research Campaign 1999

1993). Therefore, the anti-tumour effect of CPT-11 may be mediated both by SN-38 produced in the liver and transported to the tumour site by blood flow, and by SN-38 produced in the tumour cells themselves. The relative contribution of these two amounts of active drug is unknown. The importance of carboxylesterase activation of CPT-11 has suggested some strategies to enhance this local activation by in vivo transfer of a human or rabbit liver carboxylesterase c-DNA into tumours with concomitant local administration of CPT-11 (Kojima et al, 1998; Danks et al, 1998). Our study showed that the CPT-CE activity was similar in liver and in normal colon. Moreover, the activity in the normal adjacent colon mucosa was higher than in tumour in 65% of patients, in agreement with the data of Lund-Pero et al (Lund-Pero et al, 1994). The administration of CPT-11 induced several side-effects, among them acute and delayed diarrhoea. A local conversion of CPT-11 to SN-38 by the CPT-CE present in normal colon cells could be responsible of a local cytotoxic effect. Takasuma et al, 1996) evaluated the CPT-CE activity along the digestive tract and demonstrated that carboxylesterase was lower in the colon than in the ileum and jejunum. The mechanism of action of topo I inhibitors requires the presence of a DNA synthesis (Creemers et al, 1994) and cells constituting the colon epithelium have a high mitotic index. In these conditions, even a low amount of SN-38 formed could mediate a significant cytotoxicity potentially responsible for a diarrhoea.

Topo I activity is the second factor in the anti-tumour activity of CPT-11. Topo I is a cellular target of CPT-11 and the sensitivity to CPT-11 may be related to the topo I gene expression, the topo I protein levels, the activity of the enzyme, and/or the formation of drug stabilized cleavable complexes (Slichenmyer et al, 1993; Creemers et al, 1994; Tanizawa et al, 1994). Our study constitutes the most extensive series of primary colorectal tumours analysed for topo I activity ($n = 53$) and includes eight liver metastases. This activity was highly variable in tumour and normal adjacent tissue. The variability of the topo I activity in tumour was higher than that observed by McLeod et al (1994) and Husain et al (1994). It was comparable to that found by Bronstein et al (1996) in different tumour types. Our data demonstrated that topo I activity was also higher in the tumour than in adjacent normal tissue. This difference may contribute to the favourable therapeutic index of CPT-11. The ratio of tumour compared to normal topo I activity is different from that reported by Husain et al (1994) and Giovanella et al (1989) who showed, in all cases tested, an 11- to 40-fold increase in catalytic activity of topo I in tumour compared to matched normal controls, leading to the expectation of a high response rate with CPT-11. Our results showed that only some tumours had very high levels of topo I (11 of 53 patients with tumoural topo I activity upper than 40 000 U mg protein$^{-1}$) and/or very high ratio between tumour and normal tissues (12 of 53 patients with a ratio above 5). Moreover, topo I activity decreased with increasing clinical stage of disease, mainly in liver metastases in which it was significantly lower than in primary colon tumours. This observation is in contrast with the data reported by Giovanella (1989). The difference in the distribution of Dukes' stages among the population of patients studied and the determination of the topo I copy number instead of a topo I activity could explain the discrepancies between these two studies. Our study demonstrated that both the CPT-CE and the topo I activities are widely variable in the malignant and the normal tissue of patients with colorectal carcinomas. This high variability could influence the susceptibility of these tumours to CPT-11-based chemotherapy. Clinical studies are in progress in our institution to explore the correlation between tumour enzyme activities and clinical outcome in patients with metastatic colon cancer treated with CPT-11-based chemotherapy.

### REFERENCES

Benedetti P, Fiorani P, Capuani L and Wang JC (1993) Camptothecin resistance from a single mutation changing glycine 363 of human DNA topoisomerase I to cysteine. *Cancer Res* **53**: 4343–4348

Bradford MM (1976) A rapid and sensitive method for quantification of microgram quantities of protein utilizing the principle of protein-dye binding. *Anal Biochem* **72**: 248–254

Bronstein IB, Vorobyev S, Timofeev A, Jolles CJ, Alder SI and Holden JA (1996) Elevations of DNA topoisomerase I catalytic activity and immunoprotein in human malignancies. *Oncol Res* **8**: 17–25

Codegoni AM, Castagna S, Mangioni C, Scovassi AI, Broggini M and D'Incalci M (1998) DNA-topoisomerase I activity and content in epithelial ovarian cancer. *Ann Oncol* **9**: 313–319

Cornarotti M, Capranico G, Bohm S, Oriana S, Spatti GB, Mariani L, Ballabio G and Zunino F (1996) Gene expression of DNA topoisomerases I, II alpha and II beta and response to cisplatin-based chemotherapy in advanced ovarian carcinoma. *Int J Cancer* **67**: 479–484

Creemers GJ, Lund B and Verweij J (1994) Topoisomerase I inhibitors: topotecan and irenotecan. *Cancer Treat Rev* **20**: 73–96

Danks MK, Morton CL, Pawlik CA and Potter PM (1998) Overexpression of a rabbit liver carboxylesterase sensitizes human tumor cells to CPT-11. *Cancer Res* **58**: 20–22

Deffie AM, Batra JK and Goldenberg GJ (1989) Direct correlation between DNA topoisomerase II activity and cytotoxicity in adriamycin-sensitive and -resistant P388 leukemia cell lines. *Cancer Res* **49**: 58–62

Giovanella BC, Stehlin JS, Wall ME, Wani MC, Nicholas AW, Liu LF, Silber R and Potmesil M (1989) DNA topoisomerase I-targeted chemotherapy of human colon cancer in xenografts. *Science* **246**: 1046–1048

Gupta E, Lestingi TM, Mick R, Ramirez J, Vokes EE and Ratain MJ (1994) Metabolic fate of irinotecan in humans: correlation of glucuronidation with diarrhea. *Cancer Res* **54**: 3723–3725

Haaz MC, Rivory L, Riché C, Vernillet L and Robert J (1998) Metabolism of irinotecan (CPT-11) by human hepatic microsomes: participation of cytochrome P-450 3A and drug interactions. *Cancer Res* **58**: 468–472

Hosokawa M, Maki T and Satoh T (1990) Characterization of molecular species of liver microsomal carboxylesterases of several animal species and humans. *Arch Biochem Biophys* **277**: 219–227

Husain I, Mohler JL, Seigler HF and Besterman JM (1994) Elevation of topoisomerase I messenger RNA, protein, and catalytic activity in human tumors: demonstration of tumor-type specificity and implications for cancer chemotherapy. *Cancer Res* **54**: 539–546

Jansen WJM, Zwart B, Hulscher STM, Giaccone G, Pinedo HM and Boven, E (1997) CPT-11 in human colon-cancer cell lines and xenografts – characterization of cellular sensitivity determinants. *Int J Cancer* **70**: 335–340

Jaxel C, Kohn KW, Wani MC, Wall ME and Pommier Y (1989) Structure-activity study of the action of camptothecin derivatives on mammalian topoisomerase I: evidence for a specific receptor site and relation to antitumor activity. *Cancer Res* **49**: 1465–1469

Kawato Y, Furuta T, Aonuma M, Yasuoka M, Yokokura T and Matsumoto K (1991) Antitumor activity of a camptothecin derivative, CPT-11, against human tumor xenografts in nude mice. *Cancer Chemother Pharmacol* **28**: 192–198

Knab AM, Fertala J and Bjornsti MA (1993) Mechanisms of camptothecin resistance in yeast DNA topoisomerase I mutants. *J Biol Chem* **268**: 22322–22330

Kojima A, Hackett NR, Ohwada A and Crystal RG (1998) In vivo human carboxylesterase cDNA gene transfer to activate the prodrug CPT-11 for local treatment of solid tumors. *J Clin Invest* **101**: 1789–1796

Kunimoto T, Nitta K, Tanaka T, Uehara N, Baba H, Takeuchi M, Yokokura T, Sawada S, Miyasaka T and Mutai M (1987) Antitumor activity of 7-ethyl-10-[4-(1-piperidino)-1-piperidino]carbonyloxy-camptothecin, a novel water-soluble derivative of camptothecin, against murine tumors. *Cancer Res* **47**: 5944–5947

Lund-Pero M, Jeppson B and Pero R (1994) Reduced non-specific steroidal esterase activity in human malignant tumor tissue from liver, colon and breast when compared to peritumoral and normal tissue levels. *Anticancer Res* **14**: 2747–2754

© Cancer Research Campaign 1999

McKay JA, Murray GI, Weaver RJ, Even SWB, Melvin WT and Burke MD (1993) Xenobiotic metabolising enzyme expression in colonic neoplasia. *Gut* **34**: 1234–1239

McLeod HL, Douglas F, Oates M, Symonds RP, Prakash D, van der Zee AG, Kaye, SB, Brown R and Keith WN (1994) Topoisomerase I and II activity in human breast, cervix, lung and colon cancer. *Int J Cancer* **59**: 607–611

Massaad L, de Waziers I, Ribrag V, Janot F, Beaune PH, Morizet J, Gouyette A and Chabot GG (1993) Comparison of mouse and human colon tumors with regard to phase I and phase II drug-metabolizing enzyme systems. *Cancer Res* **52**: 6567–6575

Miller SB, Main AR and Rush RS (1980) Purification and physical properties of oligomeric and monomeric carboxylesterases from rabbit liver. *J Biol Chem* **255**: 7161–7167

Niimi S, Nakagawa K, Sugimoto Y, Nishio K, Fujiwara Y, Yokoyama S, Terashima, Y and Saijo N (1992) Mechanism of cross-resistance to a camptothecin analogue (CPT-11) in a human ovarian cancer cell line selected by cisplatin. *Cancer Res* **52**: 328–333

Ogasawara H, Nishio K, Kanzawa F, Lee YS, Funayama Y, Ohira T, Kuraishi Y, Isogai Y and Saijo N (1995) Intracellular carboxyl esterase activity is a determinant of cellular sensitivity to the antineoplastic agent KW-2189 in cell lines resistant to cisplatin and CPT-11. *Jpn J Cancer Res* **86**: 124–129

Potmesil M, Hsiang YH, Liu LF, Bank B, Grossberg H, Kirschenbaum S, Forlenza, TJ, Penziner A, Kanganis D, Knowles D, Traganos F and Silber R (1988) Resistance of human leukemic and normal lymphocytes to drug-induced DNA cleavage and low levels of DNA topoisomerase II. *Cancer Res* **48**: 3537–3543

Rivory LP and Robert J (1994) Reversed-phase high-performance liquid chromatographic method for the simultaneous quantitation of the carboxylate and lactone forms of the camptothecin derivative irinotecan, CPT-11, and its metabolite SN-38 in plasma. *J Chromatogr B Biomed Appl* **661**: 133–141

Rivory LP and Robert J (1995) Identification and kinetics of a beta-glucuronide metabolite of SN-38 in human plasma after administration of the camptothecin derivative irinotecan. *Cancer Chemother Pharmacol* **36**: 176–179

Rivory LP, Bowles MR, Robert J and Pond SM (1996) Conversion of irinotecan (CPT-11) to its active metabolite, 7-ethyl-10-hydroxycamptothecin (SN-38), by human liver carboxylesterase. *Biochem Pharmacol* **52**: 1103–1111

Rothenberg M, Eckardt JR, Kuhn JG, Burris HAr, Nelson J, Hilsenbeck, SG, Rodriguez GI, Thurman AM, Smith LS, Eckhardt SG, Weiss GR, Elfring, GL, Rinaldi DA, Schaaf LJ and Von-Hoff DD (1996) Phase II trial of irinotecan in patients with progressive or rapidly recurrent colorectal cancer. *J Clin Oncol* **14**, 1128–1135

Rougier P, Bugat R, Douillard JY, Culine S, Suc E, Brunet P, Becouarn Y, Ychou M, Marty M, Extra JM, Bonneterre J, Adenis A, Seitz JF, Ganem G, Namer M, Conroy T, Negrier S, Merrouche Y, Burki F, Mousseau M, Herait P and Mahjoubi M (1997) A phase II study of CPT-11 (Irinotecan) in the treatment of advanced colorectal cancer in chemotherapy-naive patients and patients treated with 5FU-based chemotherapy. *J Clin Oncol* **15**: 251–260

Satoh T and Hosokawa M (1995) Molecular aspects of carboxylesterase isoforms in comparison with other esterases. *Toxicol Lett* **82/83**: 439–455

Satoh T, Hosokawa M, Atsumi R, Suzuki W, Hakusui H and Nagai E (1994) Metabolic activation of CPT-11, 7-ethyl-10-[4-(1-piperidino)-1-piperidino]carbonyloxycamptothecin, a novel antitumor agent, by carboxylesterase. *Biol Pharm Bull* **17**: 662–664

Slatter JG, Su P, Sams JP, Schaaf LJ and Wienkers LC (1997) Bioactivation of the anticancer agent CPT-11 to SN-38 by human hepatic microsomal carboxylesterases and the in vitro assessment of potential drug interactions. *Drug Metab Disp* **25**: 1157–1164

Slichenmyer WJ, Rowinsky EK, Donehower RC and Kaufmann SH (1993) The current status of camptothecin analogues as antitumor agents. *J Natl Cancer Inst* **85**: 271–291

Smith PK, Krohn RI, Hermanson GT, Mallia AK, Gartner FH, Provenzano MD, Fijimoto EK, Goeke NM, Olson BJ and Klenk DC (1985) Measurement of protein using bicinchoninic acid. *Anal Biochem* **150**: 76–85

Takano H, Kohno K, Matsuo K, Matsuda T and Kuwano M (1992) DNA topoisomerase-targeting antitumor agents and drug resistance. *Anticancer Drugs* **3**: 323–330

Takasuna K, Hagiwara T, Hirohashi M, Kato M, Nomura M, Nagai E, Yokoi T and Kamataki T (1996) Involvement of β-glucuronidase in intestinal microflora in the intestinal toxicity of the antitumor camptothecin derivative irinotecan hydrochloride (CPT-11) in rats. *Cancer Res* **56**: 3752–3757

Tanizawa A, Fujimori A, Fujimori Y and Pommier Y (1994) Comparison of topoisomerase I inhibition, DNA damage, and cytotoxicity of camptothecin derivatives presently in clinical trials. *J Natl Cancer Inst* **86**: 836–842

Tsuji T, Kaneda N, Kado K, Yokokura T, Yoshimoto T and Tsuru D (1991) CPT-11 converting enzyme from rat serum: purification and some properties. *J Pharmacobiodyn* **14**: 341–349

van Ark-Otte J, Kedde MA, van der Vijgh WJF, Dingemans A-MC, Jansen WJM, Pinedo HM, Boven E and Giaccone G (1998) Determinants of CPT-11 and SN-38 activities in human lung cancer cells. *Br J Cancer* **77**: 2171–2176

van der Zee AG, de Jong S, Keith WN, Hollema H, Boonstra H and de Vries EG (1994) Quantitative and qualitative aspects of topoisomerase I and II alpha and beta in untreated and platinum/cyclophosphamide treated malignant ovarian tumors. *Cancer Res* **54**: 749–755

 *© Cancer Research Campaign 1999*
